# Otx2 enhances transdifferentiation of Müller cells‐derived retinal stem cells into photoreceptor‐like cells

**DOI:** 10.1111/jcmm.13995

**Published:** 2018-11-19

**Authors:** Yu Xiong, Hongpei Ji, Zhipeng You, Fei Yao, Rongrong Zhou, Weitao Song, Xiaobo Xia

**Affiliations:** ^1^ Department of Ophthalmology Xiangya Hospital Central South University Changsha China; ^2^ Department of Ophthalmology The Second Affiliated Hospital of Nanchang University Nanchang China; ^3^ Department of Oncology Xiangya Hospital Central South University Changsha China

**Keywords:** Müller cells, Otx2, photoreceptor cells, stem cells, Wnt signalling

## Abstract

Retinal Müller glial cells have the potential of neurogenic retinal progenitor cells, and could reprogram into retinal‐specific cell types such as photoreceptor cells. How to promote the differentiation of Müller cells into photoreceptor cells represents a promising therapy strategy for retinal degeneration diseases. This study aimed to enhance the transdifferentiation of rat Müller cells‐derived retinal stem cells (MC‐RSCs) into photoreceptor‐like cells and explore the signalling mechanism. We dedifferentiated rat Müller cells into MC‐RSCs which were infected with Otx2 overexpression lentivirus or control. The positive rate of photoreceptor‐like cells among MC‐RSCs treated with Otx2 overexpression lentivirus was significantly higher compared to control. Furthermore, pre‐treatment with Crx siRNA, Nrl siRNA, or GSK‐3 inhibitor SB‐216763 reduced the positive rate of photoreceptor‐like cells among MC‐RSCs treated with Otx2 overexpression lentivirus. Finally, Otx2 induced photoreceptor precursor cells were injected into subretinal space of *N*‐methyl‐*N*‐nitrosourea induced rat model of retinal degeneration and partially recovered retinal degeneration in the rats. In conclusion, Otx2 enhances transdifferentiation of MC‐RSCs into photoreceptor‐like cells and this is associated with the inhibition of Wnt signalling. Otx2 is a potential target for gene therapy of retinal degenerative diseases.

## 1 INTRODUCTION

Retinal degenerative diseases (RDDs) include age‐related macular degeneration (AMD), retinitis pigmentosa (RP), Leber's congenital amaurosis (LCA) and cone‐rod dystrophy (CRD), and affect millions of people worldwide.^1^ RDDs are generally caused by the loss of photoreceptors and lead to irreversible blindness.^2^, ^3^ Therefore, stem cells replacement therapies that aim to replace lost photoreceptors have attracted considerable attention.^4^, ^5^ Recent studies suggest that four types of stem cells could provide the sources for photoreceptors: embryonic stem cells (ESCs), bone marrow mesenchymal stem cells (BMSCs), induced pluripotent stem cells (iPSCs) and autologous retinal stem cells.^6^, ^7^, ^8^ Although ESCs, iPSCs or BMSCs offer the possibility of specific differentiation into photoreceptor cells, they have a variety of problems at present, mostly because of complex procedures, graft rejection and potential oncogenesis. On the other hand, ESCs remain highly controversial of ethical issues. Therefore, autologous retinal stem cell therapy has a significant advantage and potential. However, retinal stem cells only exist in the ciliary margin zone (CMZ) and retinal pigmented epithelium (RPE), and are far short of clinical need.^9^ Accordingly, it is urgent to develop novel methods to generate substantial photoreceptor cells that can be integrated into the injured retina.

Müller glia cells are the major cell type in the mammalian retina and contribute to the maintenance of retinal homeostasis and trophic support for retinal neurons.^10^ In acutely injured retinas, Müller glial cells can proliferate and reprogram into a neural progenitor state, which then differentiate into retinal neurons, but the efficiency is low. The reprogrammed progenitors express transcription factors similar to embryonic retinal progenitors such as Chx10, Pax6, Sox2, Sox9, Six3 and Ascl1a.^11^ Previous studies have demonstrated that Müller glia cells proliferate in vitro to form neurosphere which can be differentiated into retinal‐specific cell types, including retinal ganglion cells, bipolar neurons and rod photoreceptors.^12^, ^13^ However, the induce photoreceptor cells are too few to replace the lost or injured cells. Thus, how to promote the differentiation of Müller cells into photoreceptor cells represents a promising therapy strategy for RDDs.

The differentiation of retinal stem cells involves several transcription factors. Among them, orthodenticle homeobox 2 (Otx2) is a crucial transcription factor for photoreceptor cell specification,^14^ it regulates the expression of cone‐rod homeobox (Crx) by binding to Crx promoter,^15^ and then both of them act on the downstream target gene Nrl and promote the generation of rhodopsin.^16^, ^17^, ^18^ Wnt signalling is known to be crucial for the renewal of retinal progenitor cells and the maintenance of steady‐state of stem cells.^19^ Wnt signalling is negatively regulated by Otx2 in the midbrain dopaminergic neural stem cells.^20^

In this study we constructed lentivirus pGC‐FU‐Otx2‐EGFP and overexpressed Otx2 in rat Müller cells‐derived retinal stem cells (MC‐RSCs), to induce the differentiation into photoreceptor‐like cells. Furthermore, we treated MC‐RSCs with Crx siRNA, Nrl siRNA or GSK‐3 inhibitor SB‐216763, and examined their effects on Otx2 induced differentiation into photoreceptor‐like cells. Finally, Otx2 induced photoreceptor precursor cells were injected into subretinal space of *N*‐methyl‐*N*‐nitrosourea (MNU) induced rat model of retinal degeneration to test the in vivo efficacy of Otx2 induced photoreceptor‐like cells to recover retinal degeneration.

## 2 MATERIALS AND METHODS

### 2.1 Ethics statement

All animal experiments were performed following the Association for Research in Vision and Ophthalmology (ARVO) statements for the Use of Animals in Ophthalmic and Vision Research, and the procedures were approved by the Animal Research Committee of Xiangya School of Medicine, Central South University (SYXK 2015‐0017).

### 2.2 Primary culture and dedifferentiation of retinal Müller cells

Retinal Muller cells were isolated from SD rats and dedifferentiated into retinal stem cells as described in detail in our previous study.^21^

### 2.3 Construction and infection of Lentivirus pGC‐FU‐Otx2‐EGFP

Lentivirus pGC‐FU‐Otx2‐EGFP was constructed by GENECHEM (Shanghai, China). Müller cells‐derived neurospheres were dissociated into single cells and the cells were infected at the multiplicity of infection (MOI) of 10. Neurospheres infected by pGC‐FU‐EGFP lentivirus were negative control. Twenty‐four hours after infection, the cells were harvested, washed and then cultured in 1 ml differentiation Neurobasal‐A medium (Gibco, Grand Island, NY, USA) supplemented with retinoic acid (RA, 1 μmol/L, Sigma) and taurine (100 μmol/L, Sigma) at 37°C in a 5% CO_2_ incubator, the medium was changed every 2‐3 days. After 1 week culture, the cells were plated onto 0.01% poly‐d‐lysine (Sigma) coated 24‐mm coverslips (Corning) at a concentration of 1 × 10^4^ cells/well. After further culture for 7 or 14 days, the cells were fixed by cold 4% paraformaldehyde for immunofluorescence staining to calculate the percentage of rhodopsin positive cells.

### 2.4 Pre‐treatment with siRNAs and GSK3 inhibitor

Purified neurospheres were dissociated with Accutase (Sigma, St. Louis, MO, USA), and 1 × 10^5^ cells/well in 6‐well plates were transfected with Silencer^®^ Select si‐Crx RNA (s133418), Silencer^®^ Select si‐Nrl RNA (s14799) or Silencer^®^ Select Negative Control siRNA (4390844) (all from Ambion Inc, Austin, TX, USA), performed with Lipofectamine RNAiMAX reagent (Invitrogen) according to the manufacturer's instructions, or were treated with GSK3 inhibitor SB‐216763. The cells were cultured in 2 ml differentiation Neurobasal‐A medium supplemented with RA (1 μmol/L) and taurine (100 μmol/L). After 72 hours, the cells were infected with pGC‐FU‐Otx2‐EGFP or empty lentivirus. The cells were harvested 7 days later and analysed by immunofluorescence staining to calculate the percentage of rhodopsin positive cells.

### 2.5 *N*‐methyl‐*N*‐nitrosourea (MNU) treated photoreceptor degeneration rat model

MNU (Aladdin; N136701, Shanghai, China) was stored at −20°C in the dark and dissolved into saline containing 0.05% acetic acid immediately prior to use. Female Sprague‐Dawley rats (6 weeks old) were treated with ip, dose of sterile MNU (60 mg/kg body weight) as described previously.^22^ Rats were maintained at 22 ± 2°C and 60 ± 10% humidity with a 12:12 hours light/dark cycle with free access to the food and water. Rats were anaesthetized by intraperitoneal injection of sodium pentobarbital, the pupils were dilated with tropicamide eye drops (Santen, Japan), and corneas were anaesthetized with 0.5% proparacaine (Alcon, Forth Worth, TX, USA) if necessary. The rats were randomly divided into three groups (n = 6): Group A received no further treatment and served as model group; Group B received subretinal injection of lentivirus pGC‐FU‐EGFP infected retinal stem cells and Group C received subretinal injection of lentivirus pGC‐FU‐Otx2‐EGFP infected retinal stem cells. For subretinal injection, retinal stem cells were differentiated in RA + taurine Neurobasal‐A medium for 3 days. The success of photoreceptor differentiation was confirmed by Rhodopsin immunostaining on day 4, and differentiated cells were dissociated into single cells at a concentration of 1 × 10^5^ cells/μL at day 3. Anaesthetized rats received the injection of 2.5 μL of cell suspension by subretinal route under an operating microscope (Zeiss OPMI Pico; Carl Zeiss Meditec GmbH), the rat fundus could be visualized with the application of a drop of 2.5% methylcellulose and covered with a glass microscope slide to the eye. Subretinal bleb was observed after the injection under operating microscope to make sure that there was no retinal bleeding.

### 2.6 Hematoxylin and eosin staining

Rats were killed 1, 3, 5 and 7 days after injection, and their eyeballs were enucleated immediately. The eyes were fixed overnight in 60% methanol, 30% chloroform and 10% acetic acid, paraffin embedded and cut into 4 μm sections parallel to the optic axis and nerve (including the ora serrata). The sections were stained with haematoxylin and eosin (H&E) and observed under optical microscope (Olympus BX60, Hamburg, Germany). The central and peripheral thickness of the total retina and the outer nuclear layer (ONL) were measured at two sites of 0.3 mm and 0.8 mm from the centre of the optic disk with Image‐Pro Plus 5.0 software (Media Cybernetics, Silver Spring, MD, USA).

### 2.7 Immunofluorescence analysis

Rat retinal tissue sections or retinal cells were fixed with 4% paraformaldehyde for 15 minutes at room temperature and permeabilized with PBS containing 3% bovine serum albumin (BSA), 5% goat serum and 0.3% Triton X‐100 at 37°C for 1 hour. The samples were incubated overnight at 4°C with the primary antibodies (listed in Table 1). After washing, the samples were incubated in the dark at room temperature for 2 hours with Alexa Fluor‐conjugated secondary antibodies (Invitrogen or Molecular Probes). For negative controls PBS was used instead of primary antibody. After immunostaining, the samples were counterstained with DAPI. Six randomly selected fields from each sample were observed and fluorescent signals were captured under fluorescent microscopy (Leica DMI4000B, Solms, Germany).

**Table 1:** The antibodies used in this study

Antigen	Type	Dilution	Source
Rhodopsin	Mouse monoclonal	1 in 1000(IF)	Sigma
Synaptophysin	Mouse monoclonal	1 in 20(IF)	Abcam
Otx2	Rabbit polyclonal	1 in 1000(WB)	Abcam
Crx	Mouse polyclonal	1 in 2000(WB)	Sigma
Nrl	Rabbit monoclonal	1 in 1500(WB)	Abcam
Dkk‐1	Rabbit monoclonal	1 in 1000(WB)	CST
β‐catenin	Mouse monoclonal	1 in 2000(WB)	CST
GAPDH	Mouse monoclonal	1 in 3000(WB)	Proteintech

IF, Immunofluorescent; WB, Western blot analysis.

### 2.8 qRT‐PCR

Total RNA was extracted from retinal cells or retinal tissue performed with Trizol reagent (Ambion, Austin, TX, USA) and treated with RNase‐free DNase I. Reverse transcription was performed with the PrimeScript RT kit with gDNA Eraser (Takara, Tokyo, Japan). PCR was performed with the SYBR Premix Ex Taq (Takara) and the primers listed in Table 2. Glyceraldehyde phosphate dehydrogenase (GAPDH) was an internal standard. Experiments were performed at least in triplicate. Target mRNA levels were quantified using the comparative threshold cycle (Ct) method, fold change = 2^ΔΔCt^, ΔΔCt = (Ct_Target_ − Ct_GAPDH_)_sample_ − (Ct_Target_ − Ct_GAPDH_)_control_.

**Table 2:** Primers used in this study

Gene	Primer sequence	Product size (bp)	Annealing temp (°)	Acc. No
Otx2	Forward: 5′‐ACCAGCCACCTCAATCAGTC‐3′ Reverse: 5′‐TTCCAAGAGGCAGTTTGGTC‐3′	119	59	NM_001100566.1
Crx	Forward: 5′‐TATATGAACCCGGGACCTCA‐3′ Reverse: 5‐′CCTCACGTGCATACACATCC‐3′	202	60	AB021129.1
Nrl	Forward: 5′‐GAAATAAAGCGGGAGCCTTC‐3′ Reverse: 5′‐GTGGCCAGCCAGTATAGCTC‐3′	170	58	NM_001106036.2
Dkk‐1	Forward: 5′‐ACAGCCTAAATGCGATGGAC‐3′ Reverse: 5′‐CAGGGGAGTTCCATCAAGAA‐3′	172	60	NM_001106350
GAPDH	Forward: 5′‐AGACAGCCGCATCTTCTTGT‐3′ Reverse: 5′‐CTTGCCGTGGGTAGAGTCAT‐3′	246	60	NM_017008.4

### 2.9 Western blot analysis

Proteins were extracted from the cells or retinal tissues by using Radio Immuno Precipitation Assay (RIPA, Millipore, Bedford, MA, USA) buffer containing 1:100 protease inhibitor cocktail (Sigma). The protein concentration was determined by using a microplate reader. Lysates were separated on SDS‐PAGE and transferred onto polyvinylidene fluoride (PVDF) membranes (Millipore). The membranes were blocked for 1 hour with 5% nonfat milk in 1 ×TBS containing 0.1% Tween 20 and then incubated overnight at 4°C with primary antibodies listed in Table 1. After several washes, the membranes were incubated with HRP‐conjugated secondary antibodies (Gibco) at room temperature for 1 hour. The signal was visualized using Pierce ECL kit (Thermo Scientific). The blots were visualized on X‐Ray Film and quantified using Image J software (NIH). GAPDH was used as loading control.

### 2.10 Electroretinograms (ERG)

The rats were dark adapted for 2 hours, anaesthetized with ip, injection of 10% chloral hydrate and the pupils were dilated with 1% tropicamide and treated with a topical anaesthetic (0.5% proparacaine). A silver‐impregnated wire loop record electrodes were mounted on the surface of the cornea. A reference electrode was placed behind the ears and a ground electrode was placed under the cheek to detect the electroretinogram of the rats. Hydroxypropyl methylcellulose was applied to maintain corneal hydration. The measurements followed the International Society for Clinical Electrophysiology of Vision (ISCEV) standardization protocol.

### 2.11 Statistical analysis

All data were presented as mean ± SD. Comparison between groups was assessed by unpaired Student's *t*‐test, and the differences among multiple groups were analysed by analysis of variance (ANOVA). All statistical tests were performed using SPSS Statistics 22.0 for Windows (SPSS Inc., Armonk, NY, USA). *P *< 0.05 was considered significant.

## 3 RESULTS

### 3.1 Otx2 enhances the differentiation of MC‐RSCs into photoreceptor‐like cells

**Figure 1:**
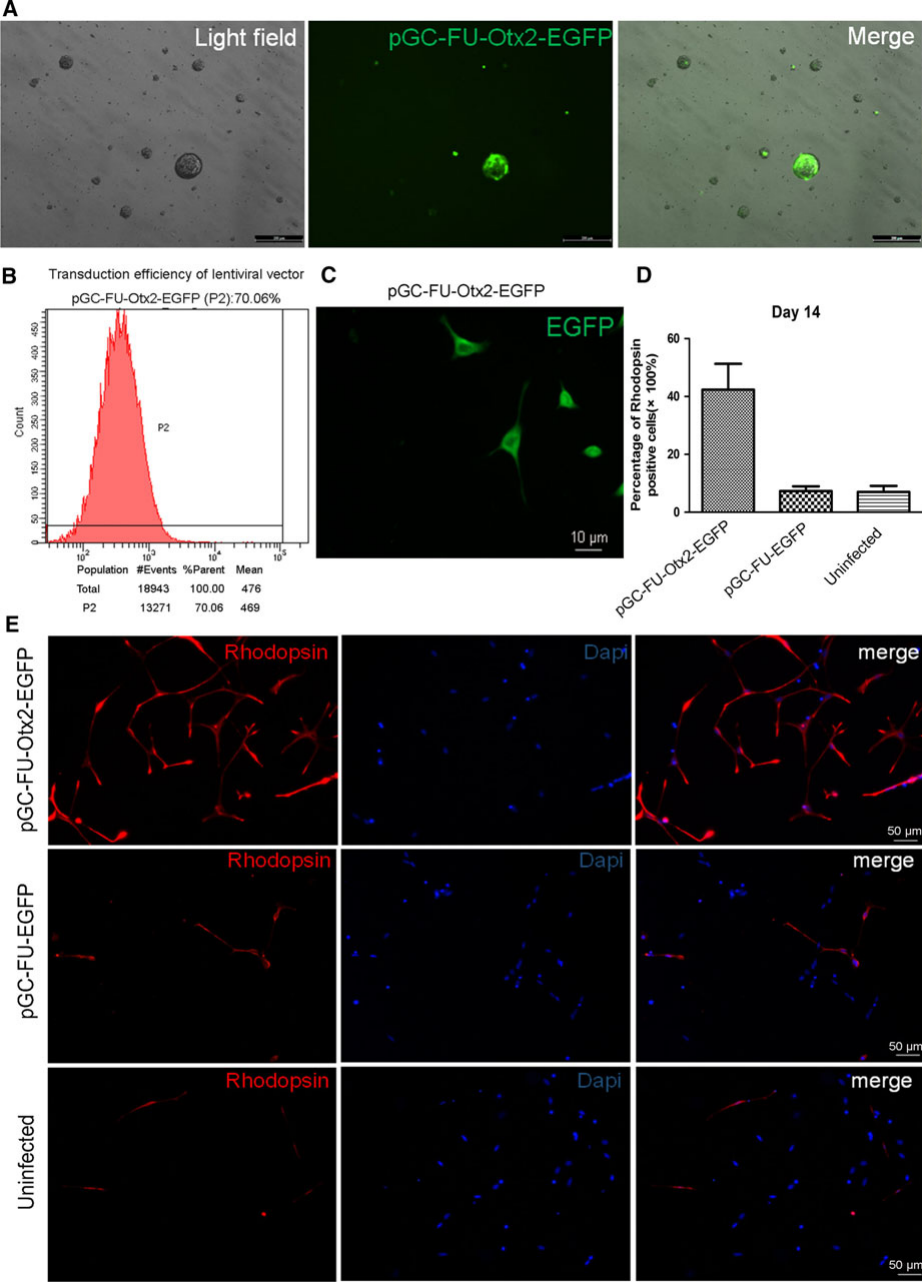
Transduction of MC‐RSCs by lentivirus. A, 48 hours after infection with pGC‐FU‐Otx2‐EGFP lentivirus, we observed strong green fluorescence in the cytoplasm of MC‐RSCs (bar = 200 μm). B, Flow cytometry analysis showed that transduction efficiency of pGC‐FU‐Otx2‐EGFP lentivirus was 70.06% in MC‐RSCs. C, 72 hours after infection with pGC‐FU‐Otx2‐EGFP lentivirus, the differentiated MC‐RSCs still showed strong green fluorescence, and the distribution was even (bar = 10 μm). D, At 7 days after differentiation, immunofluorescence staining showed that differentiated photoreceptor‐like cell bodies were elongated, cytoplasm was rich, axons were long and extended and the nucleus was located in the centre of the cell body. Rhodopsin was stained red and the nucleus was stained blue (bar = 50 μm). E, The percentage of cells positive for Rhodopsin in pGC‐FU‐Otx2‐EGFP group (group A) (42.33 ± 8.96%) was significantly higher than that of pGC‐FU‐EGFP group (group B) (7.32 ± 1.67%) and uninfected group (group C) (7.01±2.09%) on Day 14. Data were presented as mean ± SD (n = 3)

We obtained and characterized MC‐RSCs using our previously established protocols.^21^ Next, MC‐RSCs were infected with pGC‐FU‐Otx2‐EGFP lentivirus or control pGC‐FU‐EGFP lentivirus. At 48 hours after infection, the number of EGFP cells increased and fluorescence intensity enhanced, green fluorescence was distributed homogeneously in the cytoplasm (Figure 1A). At 72 hours after infection, based on EGFP fluorescence the infection efficiency was estimated as 70.06% at multiplicity of infection (MOI) of 10 (Figure 1B). At this time, MC‐RSCs infected with pGC‐FU‐Otx2‐EGFP lentivirus grew radially, began to differentiate and continued to express GFP (Figure 1C). In contrast, MC‐RSCs without infection (blank group) had no GFP fluorescence.

Fluorescence microscopy showed that photoreceptor‐like cells were stained as red based on photoreceptor cell‐specific marker Rhodopsin. The photoreceptor‐like cells in pGC‐FU‐Otx2‐EGFP group appeared on day 4, reached the peak on day 7 and then gradually reduced. They were elongated, the cytoplasm was rich, the axons were long and the nuclei were located in the centre of the cell body (Figure 1D). At 7 days after differentiation, the positive rate of Rhodopsin in pGC‐FU‐Otx2‐EGFP group (42.33 ± 8.96%) was significantly higher than that of pGC‐FU‐EGFP group (7.32 ± 1.67%) and blank group (7.01 ± 2.09%) and there was no significant difference between blank group and pGC‐FU‐EGFP group (*P *> 0.05) (Figure 1E).

**Figure 2:**
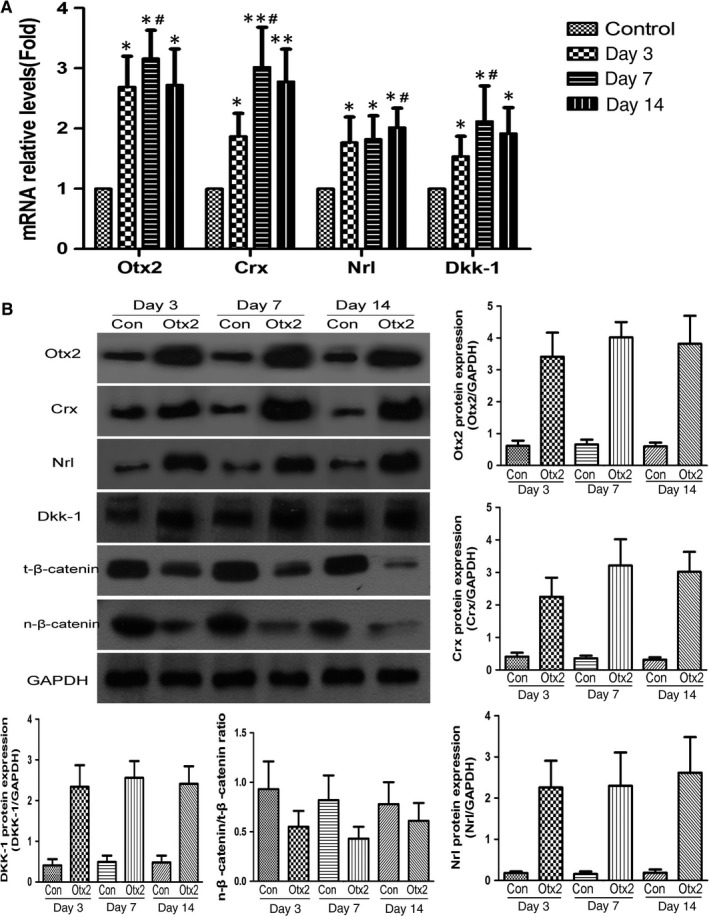
The expression of Otx2, Crx, Nrl and Dkk‐1 in MC‐RSCs transduced by lentivirus. A, qRT‐PCR analysis of Otx2, Crx, Nrl and Dkk‐1 mRNA levels in pGC‐FU‐EGFP lentivirus infected MC‐RSCs (control) and pGC‐FU‐Otx2‐EGFP lentivirus infected MC‐RSCs on day 3, 7 and 14 after infection. B, Western blot analysis of Otx2, Crx, Nrl, Dkk‐1 and total β‐catenin and nuclear β‐catenin protein levels in pGC‐FU‐EGFP lentivirus infected MC‐RSCs (Con) and pGC‐FU‐Otx2‐EGFP lentivirus infected MC‐RSCs (Otx2) on day 3, 7 and 14 after infection. GAPDH was loading control. Data were presented as mean ± SD (n = 3). *P < 0.05, **P < 0.01 vs control; ^#^ *P *< 0.05 vs day 3 or day 7

qRT‐PCR and Western blot analysis showed that in pGC‐FU‐Otx2‐EGFP group Otx2, Crx and Dkk‐1 mRNA and protein levels increased and reached the peak on day 7, while Nrl mRNA and protein levels gradually increased and reached the peak on day 14. In contrast, nuclear β‐catenin/total β‐catenin ratio (the indicator of Wnt/β‐catenin pathway activation) gradually decreased (Figure 2A,B). These data suggest that Otx2 up‐regulates the expression of Crx, Nrl and inhibits Wnt pathway during the differentiation of MC‐RSCs.

### 3.2 Crx, Nrl and Wnt signalling regulate the differentiation of MC‐RSCs into photoreceptor‐like cells

**Figure 3:**
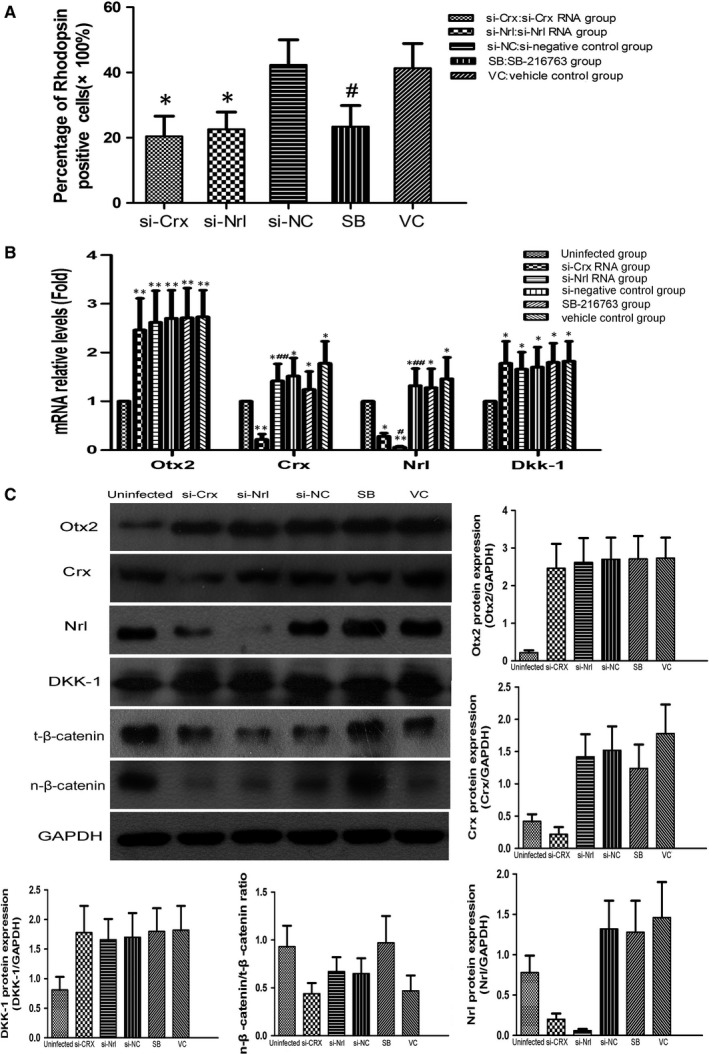
Treatment of MC‐RSCs by siRNAs or GSK3 inhibitor. A, The percentage of photoreceptor‐like cells in si‐Crx group (20.43 ± 6.21%) and si‐Nrl group (22.56 ± 5.31%) were significantly lower than in si‐NC group (42.31 ± 7.69%), *P < 0.05. The percentage of photoreceptor‐like cells in SB (23.42 ± 6.44%) was significantly lower than in VC group (41.31±7.58%), ^#^ *P *< 0.05. B and C, PCR and Western blot analysis showed that si‐Crx significantly decreased Crx and Nrl mRNA and protein levels, si‐Nrl significantly decreased Nrl mRNA and protein levels, while SB‐216763 increased nuclear β‐catenin/total β‐catenin ratio. Data were presented as mean ± SD (n = 3).*P < 0.05, **P < 0.01 vs uninfected group; ^#^ *P *< 0.05, ^##^ *P *< 0.01 vs VC group

MC‐RSCs were treated with Crx or Nrl siRNA or GSK3 inhibitor SB‐216763, qRT‐PCR and Western blot analysis showed that si‐Crx significantly reduced Crx and Nrl mRNA and protein levels, si‐Nrl significantly reduced Nrl mRNA and protein levels, while SB‐216763 increased nuclear β‐catenin/total β‐catenin ratio. The positive rate of photoreceptor‐like cells in Crx or Nrl siRNA group was 20.43 ± 6.21% and 22.56 ± 5.31%, respectively, significantly lower than NC siRNA group (42.31 ± 7.69%) (*F* = 26.99, *P *< 0.05). In contrast, the positive rate of photoreceptor‐like cells in SB‐216763 treated group (23.42 ± 6.44%) was significantly lower than vehicle treated group (41.31 ± 7.58%) (*t*=3.593, *P *< 0.05) (Figure 3A‐C).

### 3.3 Transplantation of differentiated MC‐RSCs into MNU‐induced retinal photoreceptor degeneration rat model

**Figure 4:**
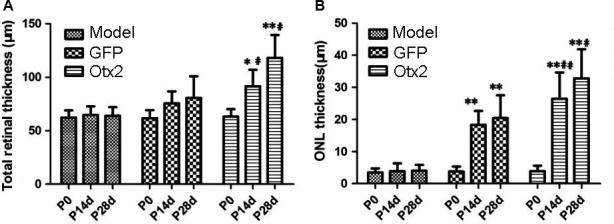
The thickness of total retina and outer nuclear layer in MNU rat model after cell transplantation. Model group received no treatment; GFP group received subretinal injection of lentivirus pGC‐FU‐EGFP infected retinal stem cells; and Otx2 group received subretinal injection of lentivirus pGC‐FU‐Otx2‐EGFP infected retinal stem cells. A, The total thickness of retina at 14 days and 28 days after subretinal injection in Otx2 group was 91.62 ± 15.27 μm and 118.06 ± 21.42 μm, respectively, significantly thicker than that of P0 (63.21 ± 6.92 μm). The total thickness of retina in Model group and GFP group had no obvious change. B, The thickness of the retinal outer nuclear layer (ONL) at 14 days and 28 days after subretinal injection was 26.44 ± 8.21 μm and 32.81 ± 9.06 μm, respectively, significantly thicker than that of P0. Data were presented as mean ± SD (n = 6).*P < 0.05, **P < 0.01 vs group A; ^#^ *P *< 0.05, ^##^ *P *< 0.01 vs group B

Photoreceptor degeneration rat model was established and validated by intraperitoneal injection of 60 mg/Kg MNU as previously described.^22^ After subretinal injection of differentiated MC‐RSCs into photoreceptor degeneration rat model, the total thickness of retina at 14 days and 28 days in Otx2 group was 91.62 ± 15.27 μm and 118.06 ± 21.42 μm, respectively, significantly thicker than that of the time of injection (63.21 ± 6.92 μm), while the total thickness of retina in model group and GFP group had no obvious change (Figure 4A).

The thickness of ONL at 14 days and 28 days after subretinal injection in Otx2 group was 26.44 ± 8.21 μm and 32.81 ± 9.06 μm, respectively, significantly thicker than that of the time of injection (3.89 ± 1.72 μm) (*P* < 0.01). These data indicate that subretinal injection of Otx2‐induced photoreceptor precursor cells promoted retinal regeneration. The thickness of ONL in GFP group was 18.32 ± 4.33 μm and 20.42 ± 7.12 μm, respectively, at 14 days and 28 days after injection, significantly thicker than that of the time of injection (3.78 ± 1.58 μm) (*P* < 0.01) (Figure 4B). There was no significant change in the thickness of ONL and total retina in Model group before and after injection (*P *> 0.05), indicating that the differentiation medium could not induce the photoreceptor cells regeneration in vivo.

### 3.4 Migration, integration and differentiation of MC‐RSCs in the host retina

**Figure 5:**
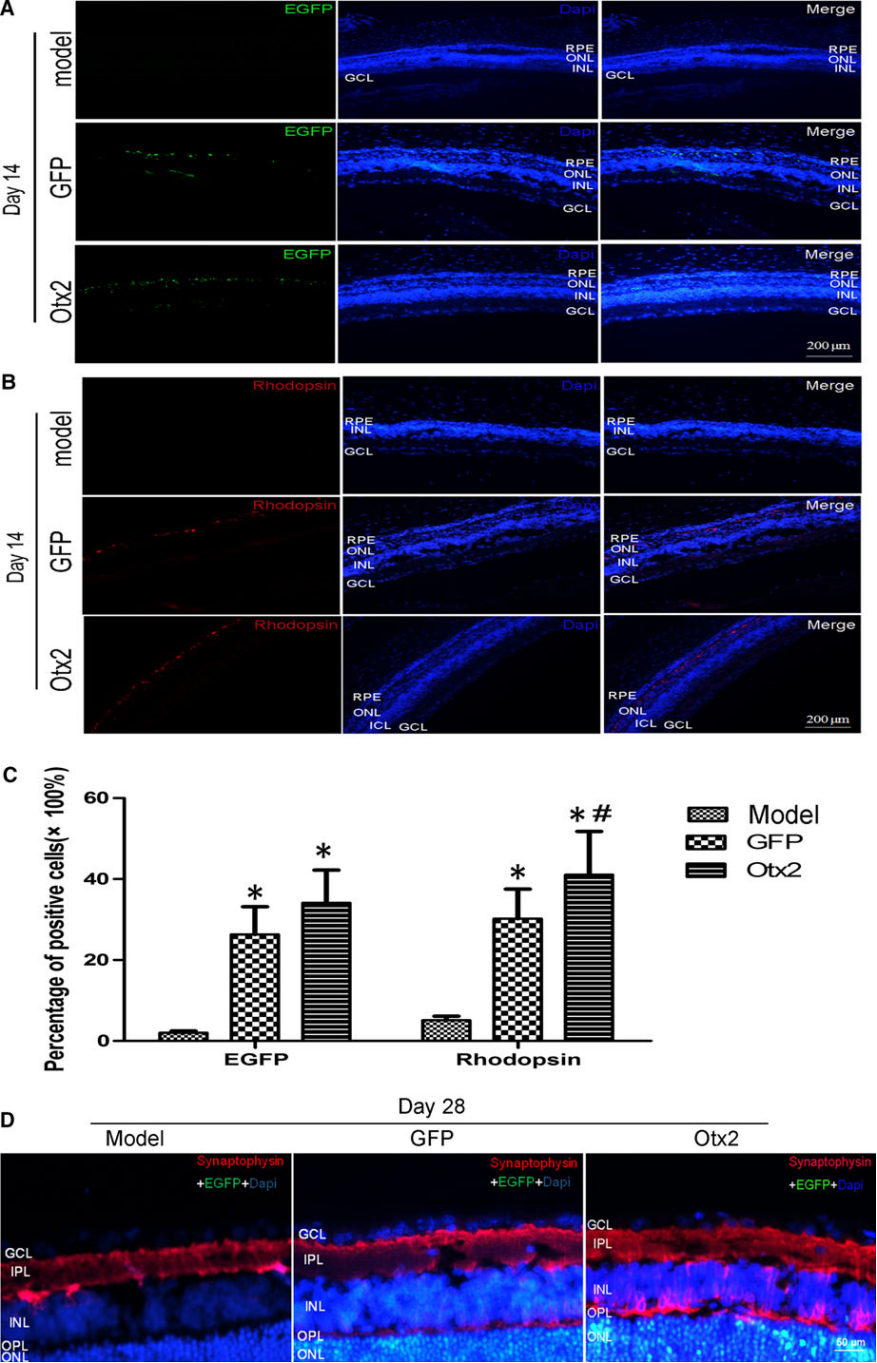
The differentiation, integration and apoptosis of transplanted cells in the host retina. Model group received no further treatment; GFP group received subretinal injection of lentivirus pGC‐FU‐EGFP infected retinal stem cells and Otx2 group received subretinal injection of lentivirus pGC‐FU‐Otx2‐EGFP infected retinal stem cells. A, 14 days after subretinal injection, GFP‐labelled cells mostly migrated to the outer nuclear layer. B, Rhodopsin expression in Otx2 group (41.09 ± 10.71%) was significantly higher than that in GFP group (22.22 ± 7.33%). C, The percentages of cells positive for GFP and Rhodopsin in each group were calculated among total cells stained by DAPI. Data were presented as mean ± SD (n = 6). D, 28 days after subretinal injection, immunofluorescence staining showed that synaptophysin (red fluorescence) and GFP (green fluorescence) were positive in OPL of Otx2 group but not in OPL of Model and GFP group (bar = 50 μm). GCL, ganglion cell layer; ONL, outer nuclear layer; INL, inner nuclear layer; IPL, inner plexiform layer; OPL, outer plexiform layer. *P < 0.05 vs Model group; ^#^ *P *< 0.05 vs GFP group

**Figure 6:**
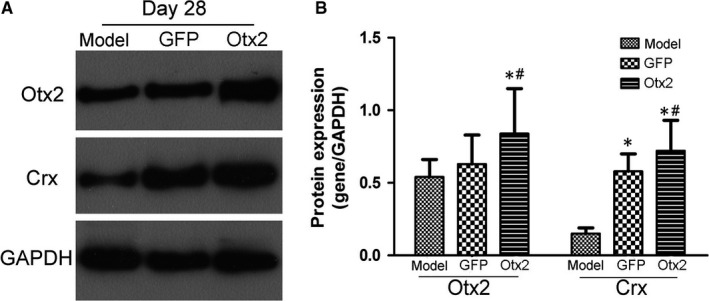
Otx2 and Crx expression after intraocular transplantation in MNU rats. Model group received no further treatment; GFP group received subretinal injection of lentivirus pGC‐FU‐EGFP infected retinal stem cells and Otx2 group received subretinal injection of lentivirus pGC‐FU‐Otx2‐EGFP infected retinal stem cells. A, Representative blots. B, Densitometry analysis showed that Otx2 and Crx protein levels in Otx2 group were significantly higher than in Model group and GFP group. Data were presented as mean ± SD (n = 6). *P < 0.05 vs Model group; ^#^ *P *< 0.05 vs GFP group

Immunofluorescence staining showed that GFP‐labelled transplanted MC‐RSCs mostly migrated to the ONL at 14 days after subretinal injection in Otx2 group and GFP group (Figure 5A). The percentage of Rhodopsin positive cells in Otx2 group was 41.09 ± 10.71%, significantly higher than that in GFP group (22.22 ± 7.33%), while almost no Rhodopsin positive cells were observed in Model group (Figure 5B,C). At 28 days after subretinal injection, immunofluorescence staining showed that synaptophysin was positive in outer plexiform layer (OPL) of Otx2 group but not in OPL of Model and GFP group (Figure 5D), indicating that photoreceptor‐like cells may establish synaptic connection with adjacent cells in Otx2 group. In addition, Otx2 and Crx protein expression levels in Otx2 group were significantly higher than in Model group and GFP group (Figure 6A,B). These data indicate that Otx2 enhances the differentiation of MC‐RSCs into photoreceptor‐like cells in MNU‐induced photoreceptor degeneration rat model.

### 3.5 Evaluation of visual function after intraocular transplantation in rats

**Figure 7:**
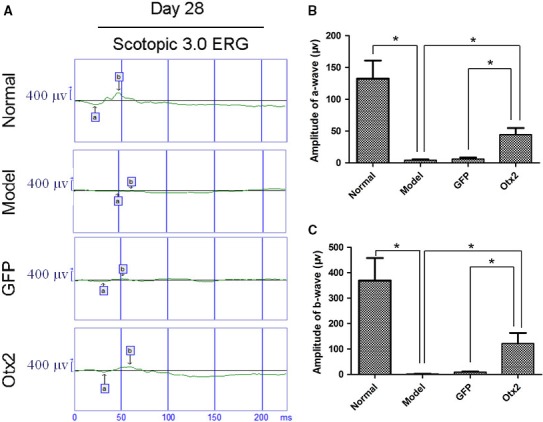
Evaluation of visual function after intraocular transplantation in MNU rats. A, Representative Scotopic 3.0 ERG of a‐ and b‐waves in healthy rats (Normal group), MNU rats (Model group), MNU rats receiving subretinal injection of lentivirus pGC‐FU‐EGFP infected retinal stem cells (GFP group) and MNU rats receiving subretinal injection of lentivirus pGC‐FU‐Otx2‐EGFP infected retinal stem cells (Otx2 group). Scotopic a‐wave (B) and b‐wave (C) in Otx2 group were higher than in Model group, but were lower than in Normal group. Data were presented as mean ± SD (n = 6). *P < 0.05

Scotopic ERG showed the deterioration of scotopic function in rats administrated with MNU, compared to healthy rats. The scotopic a‐ and b‐waves were significantly higher in MNU model rats receiving subretinal injection of lentivirus pGC‐FU‐Otx2‐EGFP infected MC‐RSCs than in MNU model rats or MNU model rats receiving subretinal injection of lentivirus pGC‐FU‐EGFP infected MC‐RSCs (Figure 7A,B). These results suggest that photoreceptor function has better recovery after transplantation of MC‐RSCs with Otx2 overexpression.

## 4 DISCUSSION

Müller cells can obtain retinal stem cell characteristics under specific conditions.^11^, ^23^ Retinal Müller cells cultured in serum‐free medium supplemented with N2 and B27 could dedifferentiate into retinal stem cells and express stem cells marker Nestin and proliferation marker Ki‐67.^21^ Transcription factors Otx2 (orthodenticle homeobox 2) and Crx (cone rod homeobox) play a key role in the development and differentiation of retinal progenitor cells into photoreceptor cells.^24^, ^25^, ^26^ Therefore, in this study we constructed lentivirus pGC‐FU‐Otx2‐EGFP and overexpressed Otx2 in rat Müller cells‐derived retinal stem cells (MC‐RSCs) to induce the differentiation into photoreceptor‐like cells. In conditional Otx2 knockout mice, the expression of Crx, Nrl, NeuroD, Blimp1 and Nr2e3 in the retina was down‐regulated while the expression of amacrine markers Glyt1 and Gad65 was up‐regulated, which are associated with the differentiation of photoreceptor cells.^27^ Koike et al found that the photoreceptor cell markers rhodopsin, M‐opsin and S‐opsin in Otx^−/−^ mice retina presented more dramatic decline than in Crx^−/−^ mice retina, further confirming the key role of Otx2 in the differentiation of photoreceptor cells.^28^

Several studies showed that Otx2 promoted the formation of photoreceptor cells by regulating the expression of downstream factors Crx and Nrl.^26^, ^27^, ^29^ Crx was the first identified specific transcription factor associated with photoreceptor cell development, which is highly expressed in mature cones and rods and controls the development and function of photoreceptor cells.^29^ Otx2 regulates the expression of Crx by binding to Crx promoter, and both of them act on the downstream target Nrl to promote the formation of rhodopsin.^24^, ^30^ In this study, in MC‐RSCs infected by pGC‐FU‐Otx2‐EGFP lentivirus, mRNA and protein expression levels of Otx2 and its downstream targets Crx and Nrl increase gradually. These data confirm that Otx2 up‐regulates the expression of Crx and Nrl in MC‐RSCs.

Otx2 promoted the differentiation of retinal stem cells by acting on its downstream gene Dkk‐1 (Dickkopf‐1), which negatively regulated Wnt pathway.^31^ Wnt pathway regulates the development of nervous system, neurogenesis, neuronal proliferation and maintenance of stable state through complex signal transduction.^32^ Dkk‐1 is an inhibitor of Wnt pathway, which can form a trimer with the receptor LRP5/6 and Kremen1/2 and block intracellular transmission of Wnt signal.^33^ Ip et al demonstrated that Dkk‐1 was the direct target of OTX2 and OTX2 bound to the H1 control element of Dkk‐1 to activate its expression.^31^ In this study, after Otx2 overexpression the mRNA and protein levels of Dkk‐1 gradually increased but total β‐catenin and nuclear β‐catenin levels decreased gradually, consistent with previous studies.^31^ Taken together, these data suggest that Otx2 could up‐regulate the expression of Dkk‐1 to inhibit Wnt pathway.

To explore signal pathway by which Otx2 enhanced the differentiation of MC‐RSCs into photoreceptor cells, we used siRNA interference and Wnt activator SB‐216763. SB‐216763 is an inhibitor of GSK‐3β, which is a key kinase to inhibit Wnt/β‐catenin pathway.^33^ Our results showed that the expression of Otx2 was not affected by si‐Crx RNA, but mRNA and protein levels of Crx and Nrl were significantly down‐regulated. The expression of Nrl mRNA and protein was significantly down‐regulated by si‐Nrl RNA treatment. On the other hand, total β‐catenin and nuclear β‐catenin levels were up‐regulated after SB‐216763 treatment, and the percentage of Rhodopsin positive cells decreased after si‐Crx RNA, si‐Nrl RNA interference or SB‐216763 treatment in MC‐RSCs infected with Otx2 lentivirus. These data suggest that Otx2 enhanced differentiation of MC‐RSCs into photoreceptor‐like cells is associated with the inhibition of Wnt pathway.

In addition, matrix environment affects the differentiation of photoreceptor cells. Osakada et al reported that in medium supplemented with Dkk‐1 (Wnt antagonist), Lefty‐A (Nodal antagonist), RA and taurine, nearly 20% ESs differentiated into Rhodopsin‐positive Rod cells.^7^ Zhong et al reported that RA and taurine could promote hiPSC differentiation into rod‐like cells in vivo and in vitro.^6^ On the basis of these data, we added RA and taurine into the differentiation medium to improve the differentiation efficiency of photoreceptor‐like cells. To evaluate the in vivo differentiation of Otx2 induced MC‐RSCs, we employed MNU‐induced photoreceptor degeneration rat model as established previously.^22^ The subretinal cavity is recognized as immune privileged region, and cell transplantation has advantages such as the low immune rejection and less systemic adverse reactions.^34^ Thus we injected pGC‐FU‐Otx2‐EGFP lentivirus infected MC‐RSCs into the subretinal space of MNU model mice, and found that Otx2 promoted retinal photoreceptor‐like cells to regenerate and thicken the outer nuclear layer.

Next we examined the localization, migration and integration of transplanted cells in the host retina. The results demonstrated that the transplanted cells could migrate to the injured retinal outer nuclear layer, differentiate into mature photoreceptor cells and integrate with the host retina. More importantly, overexpression of Otx2 could increase the differentiation efficiency of photoreceptor‐like cells in vivo. In addition, the percentage of cells with positive expression of Rhodopsin in Otx2 group was significantly higher than the percentage of cells with positive expression of EGFP, indicating that some of photoreceptor‐like cells were derived from the differentiation of transplanted cells while others may be because of the differentiation of endogenous retinal stem cells. Our observation of the spontaneous ability of MC‐RSCs to differentiate into photoreceptor‐like cells is consistent with previous report that endogenous stem cells may be activated to promote their differentiation into photoreceptor cells upon the damage of the retina.^35^

To characterize cell communication and gap junctions of photoreceptor‐like cells, we selected specific synaptic vesicle protein Synaptophysin to label the synaptic connections between regenerated photoreceptor‐like cells and adjacent neurons.^36^ Immunofluorescence assay showed that synaptophysin presented a multi‐layer distribution in the outer plexiform photoreceptor cells at the end and within the plexiform layer. These results suggest that the regenerated photoreceptor‐like cells express synaptophysin and form a synaptic neural network with the host retinal neurons to promote the reconstruction of optical path and the recovery of visual function.

Furthermore, we found that in MNU model group the amplitude of a wave decreased because of the damage of photoreceptor cells. The amplitudes of a‐ and b‐wave in Otx2 group were higher than in model group, but not reached the normal levels. These findings confirm that the transplanted cells integrated into the ONL are rod photoreceptors and form functional synaptic connection with adjacent neurons in the recipient retina to recover visual function.

In summary, Otx2 could enhance the differentiation of MC‐RSCs into photoreceptor‐like cells. Knockdown of Crx and Nrl or the activation of Wnt pathway could significantly inhibit the differentiation of MC‐RSCs into photoreceptor‐like cells in vitro. Subretinal injection of MC‐RSCs overexpressing Otx2 could promote the formation of neonatal photoreceptor‐like cells which form synaptic connection with adjacent cells, and partially rescue photoreceptor function in MNU‐treated photoreceptor degeneration rat model. Therefore, our findings provide a new avenue for photoreceptor cell regeneration to treat retinal degenerative diseases.

## CONFLICT OF INTEREST

The authors declare no conflict of interest.
